# Characterization of carbonaceous aerosol particles emitted by solid fuel combustion in rural areas

**DOI:** 10.1007/s10661-026-15514-8

**Published:** 2026-06-03

**Authors:** Sally Kheirandish, Bálint Alföldy, Péter Füri, János Osán, Árpád Farkas, Martin Rigler, Asta Gregorič, Veronika Groma

**Affiliations:** 1 Eötvös Loránd University, Budapest, 1053 Hungary; 2 Institute for Energy Security and Environmental Safety, HUN-REN Centre for Energy Research, Konkoly-Thege Miklós Út 29-33, Budapest, 1121 Hungary; 3 Aerosol d.o.o., 1000 Ljubljana, Slovenia; 4 Centre for Atmospheric Research, University of Nova Gorica, 5270 Ajdovščina, Slovenia

**Keywords:** Residential stove emission, Waste burning fingerprints, Source apportionment, Carbonaceous aerosol

## Abstract

Air pollution from waste combustion poses serious environmental and public health risks. In Hungary, illegal waste burning in residential stoves remains a significant yet underreported emission source. This study characterizes aerosols in a rapidly expanding rural settlement near Budapest (Solymár), where traffic, biomass burning, and suspected waste combustion are significant contributors to local air pollution. During a 6-week intensive winter campaign in 2025, real-time particle number concentrations, size distributions, and black carbon (BC) levels were measured using an Optical Particle Counter (Grimm 1.109) and a Portable Aethalometer (AE42). Complementary PM_2.5_ samples were collected on quartz filters over 24-h intervals and analyzed using thermo-optical methods to quantify organic (OC) and elemental carbon (EC) fractions. Video surveillance supported source interpretation. By separating traffic- and biomass-related aerosol contributions with the Aethalometer model and comparing them with EC and OC time series, we evaluated the suitability of EC and OC as source-specific markers and identified potential illegal waste-burning events. Extreme episodic pollution was observed, with 1-min PM_2.5_ concentrations reaching 212 μg/m^3^ and BC peaking at 23.8 μg/m^3^, far exceeding background levels at a nearby suburban station. Optimizing marker correlations yielded Ångström exponents of αFF = 1.15 and αBB = 2.2, the latter likely elevated by episodic waste-burning contributions. This interpretation is supported by concurrent increases in refractory EC2 fractions during periods deviating from typical biomass-burning patterns. These findings enhance emission inventories and support the development of targeted mitigation strategies, while ongoing work expands marker databases to improve source apportionment.

## Introduction

Waste burning contributes to both environmental degradation and human illness by releasing greenhouse gases and diverse harmful pollutants. Large international health assessments have identified outdoor air pollution as a key environmental driver of mortality across the globe (Cohen et al., 2017; Forouzanfar et al., 2015). Smoke generated by solid waste combustion contains particulate matter and toxic substances that can aggravate respiratory and cardiovascular conditions, and may also influence mortality and cognitive health (Manisalidis et al., 2020). Although the direct impact of rural waste burning on cognitive diseases remains uncertain, studies of military veterans exposed to burn pit emissions have reported elevated frequencies of neurological disorders, cognitive impairments, and various mental health disorders (Brooks et al., 2024). Additionally, an expanding body of scientific evidence links long-term exposure to polluted air with the development of neurodegenerative illnesses, including Alzheimer’s disease (Kilian and Kitazawa, 2018). Worldwide, chronic obstructive pulmonary disease (COPD) remains a major cause of death, and pollution from burning waste or solid fuels, along with smoking and other factors, contributes substantially to the development of this disease (World Health Organization, 2024a). According to the World Health Organization, exposure to pollutants from household fuels is estimated to account for nearly one-fifth (19%) of deaths related to household air pollution, primarily through chronic obstructive pulmonary disease (COPD). In low- and middle-income countries, approximately 23% of adult COPD deaths are linked to indoor air pollution from solid fuel use (World Health Organization, 2024c). Household exposure to kerosene and solid fuel smoke is also associated with around 11% of adult lung cancer deaths, representing 6% of all fatalities attributable to indoor air pollution (WHO, 2024c). COPD continues to rank among the leading causes of death globally (WHO, 2024a), while asthma and other chronic respiratory conditions can be aggravated by both indoor and outdoor air pollutants, including particulate matter and combustion by-products from solid fuels (WHO, 2024b). These findings emphasize the importance of controlling emissions from household energy sources and improving indoor air quality to reduce the burden of respiratory diseases.

One of the main concerns is related to the ultrafine particles (UFPs) for which combustion processes are among the major sources. Ultrafine particles usually represent a small fraction of the particle mass, but they are usually in high number in the smoke of combustion processes. For the UFPs, both the deposition pattern in the airways and the biokinetics (Kreyling, 2003) are different from those of the larger particles of the PM_10_. Although the epidemiological evidence for health effects of UFPs is still quite scarce, an increasing number of the studies suggest that UFPs exert a higher toxicity per mass unit than larger particles and may contribute to the development and progression of various diseases (Health Effects Institute, 2013). To decrease the detrimental health effects, such processes that generate UFPs, like waste burning should be monitored and strictly regulated.

Solid fuel combustion, including wood, coal, and biomass burning, remains a dominant source of atmospheric PM, with substantial contribution to PM_2.5_ and PM_10_ levels across Europe (Karagulian et al., 2015). Residential wood combustion, in particular, is a major contributor to wintertime air pollution, with studies reporting that it accounts for 30% of PM_2.5_ emissions in Portugal and 5–25% of PM_10_ emissions in Lombardy (Goncalves et al., 2012; Pastorello et al., 2011). These emissions are linked not only to severe health conditions, but also to environmental impacts, including reduced visibility and alterations in local weather patterns (Tiwari et al., 2015).

In addition to regulated solid fuel use, the illegal burning of municipal waste—such as treated wood, plastics, tires, and textiles—represents an under-researched yet significant source of air pollution. Across Europe, the proportion of households engaging in waste burning is generally estimated to remain below 5%, with higher prevalence typically reported in Eastern European countries (Gallup, 2024). However, detailed information on the composition of the burned waste, particularly regarding the relative contribution of legally and illegally burned materials, is only rarely available. In Hungary, it is estimated that, between 2 and 10% of households engage in waste burning, and the practice is widespread due to the absence of proper waste management infrastructure (Kantar Hoffman Company, 2020; Századvég Foundation, 2018). A representative Hungarian survey conducted in 2017 (Kantar Hoffmann Company, 2017) revealed that in Central Hungary, approximately 44% of respondents reported burning household waste at least once in recent years, with over 12% who burned plastic waste and over 11% who burned treated wood. Notably, the data suggests that while plastic burning is not extremely widespread, it does occur in a significant proportion of households—about 9.7% on national level and 12.4% in Central Hungary. This burning tends to happen more frequently in less-than-annual intervals, with a significant portion occurring in stoves or boilers and a smaller amount burned in open areas. In addition to plastic, 45% of respondents reported burning garden waste, often in open areas. These practices contribute notably to localized air pollution, with emissions from burning waste, particularly in stoves or boilers (e.g., 81.7% for general household waste), likely spreading into nearby residential zones. These behavioral patterns are especially important when interpreting ambient pollution measurements in residential areas, where both external industrial sources and localized, informal burning activities may significantly influence air quality readings.

In 2016, each person in Europe generated about 480 kg of municipal waste, exceeding the global average of 436 kg per person (Eurostat, 2018). To tackle this challenge, the European Union, through initiatives such as the European Green Deal and the Waste Framework Directive, has introduced ambitious goals to advance a circular economy—maximizing resource recovery and reducing landfill use (European Commission, 2023). By 2025, Member States are required to recycle or prepare for reuse at least 55% of their municipal waste, according to the legally binding EU rules (European Environment Agency, 2023a). Furthermore, by 2030, the EU has set material-specific recycling targets, including at least 55% for plastic packaging, 60% for aluminum, 75% for glass, 80% for ferrous metals, and 85% for paper and cardboard (European Commission, 2023). These targets are designed to minimize environmental harm, enhance resource efficiency, and accelerate the shift toward sustainable waste management systems.

Despite improvements in recycling and waste treatment, improper disposal practices, such as household waste burning, persist and continue to pose environmental and health risks. Although not all forms of household waste burning are considered illegal (e.g., the burning of paper or untreated biomass in certain contexts), these activities can still contribute significantly to particulate emissions and air pollution. Open burning of municipal waste releases substantial quantities of particulate matter and toxic compounds, including among others polycyclic aromatic hydrocarbons (PAHs), significantly degrading air quality (Hoffer et al., 2020). This practice is especially prevalent in regions lacking adequate waste management infrastructure and is linked to increased risks of respiratory and cardiovascular diseases.

Although the environmental and health impacts of residential waste burning are considerable, this source is often underrepresented in emission inventories, and research quantifying its emission factors and overall contribution to ambient particulate matter and PAH levels is still limited. The European Union’s Zero Pollution Action Plan aims to substantially reduce air pollution, committing to regular reviews of air quality standards by 2030 in line with the latest scientific evidence, technological progress, and societal needs (European Commission, 2021). A key target of the plan is to cut premature deaths linked to air pollution by 55% by 2030. Achieving this goal will require tackling emissions at their source, including the recognition and mitigation of illegal waste combustion.

In light of these considerations, the objective of this study was to characterize aerosols and trace pollutants emitted from waste burning and wood combustion in rural areas, with a specific focus on Solymár, Hungary. Particular attention was given to identify thermal-optical and size-resolved markers indicative of waste burning within a mixture of residential biomass combustion, traffic, and coal-related emissions. By integrating high-resolution PM, black carbon (BC), and carbon fraction measurements with plume observations and comparisons to a regional background station, this aimed to evaluate the detectability and distinctiveness of local and regional emission sources. Instead of relying on conventional modeling tools, the analysis exploits the expected temporal co-variation and strong correlations among selected tracer species. The resulting source-specific signatures provide a basis for improving source apportionment in rural settings, guiding targeted mitigation strategies to reduce health risks from waste burning, wood combustion, and other dominant local emission sources.

## Measurement and data evaluation methods

### Measurement campaign

**Fig. 1:**
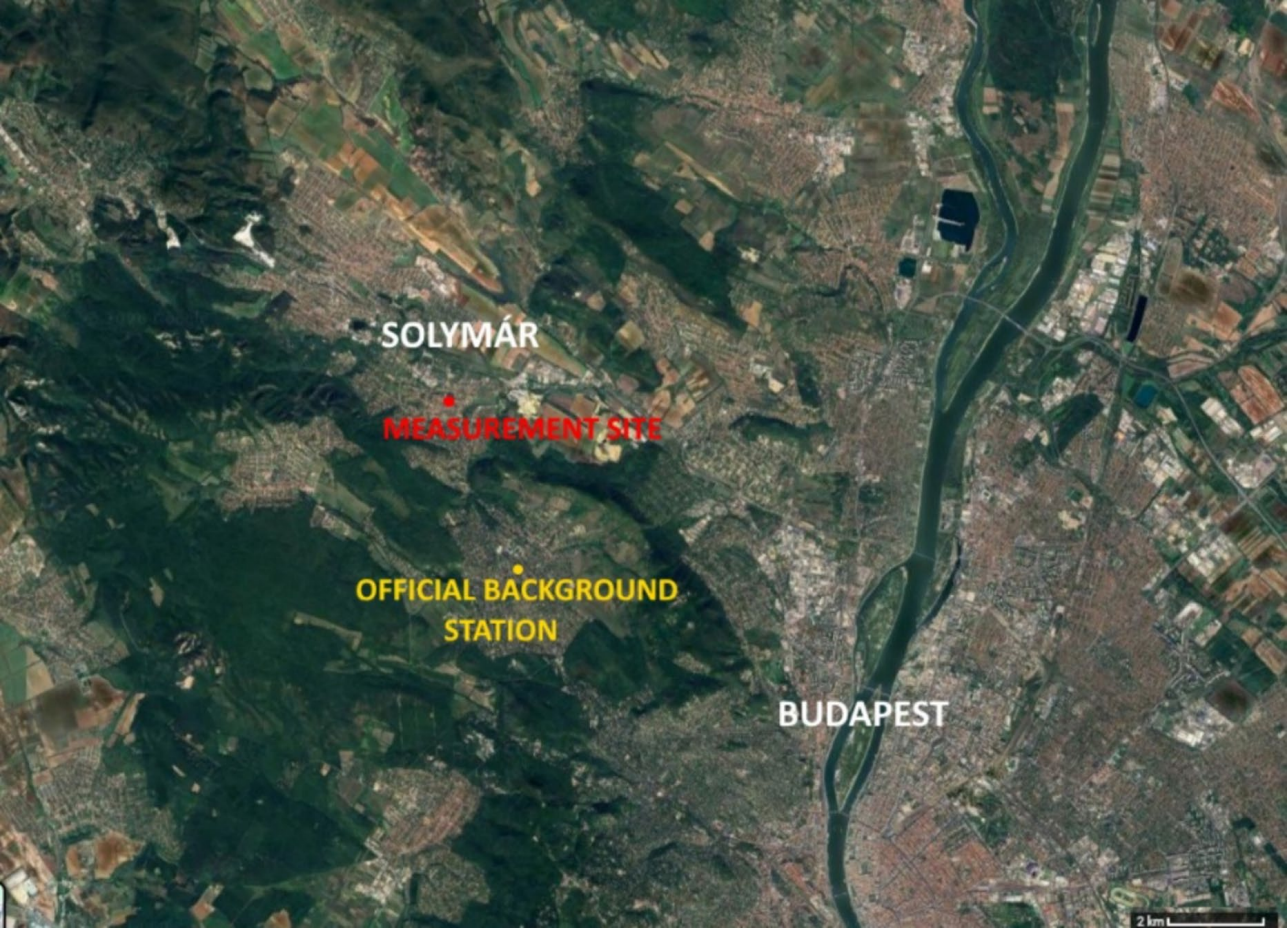
Location of the measurement site and the official background station

An intensive measurement campaign was conducted during the winter period from 10 January to 20 February 2025. The measurement site (Fig. 1) was situated in the garden of a detached family house in downtown Solymár, a small town located near Budapest, Hungary (N 47.592120, E 18.936062). Solymár is known as a typical commuter town, with a significant proportion of the population commuting daily to the capital city. As a result, the town experiences considerable vehicle traffic, including both local and transit flows. Its location within a valley further worsens air quality issues under specific meteorological conditions, such as temperature inversions or low wind speeds (HungaroMet, 2025a, 2025b). Consistent with patterns observed across rural and peri-urban regions of Hungary, residential heating practices in Solymár frequently incorporate wood combustion, either as a primary energy source or as a supplementary system alongside natural gas, largely due to its relative cost-effectiveness. The measurement site was at a distance of approximately 100 m from the town’s busiest road. Additionally, a neighboring house in close proximity is known to utilize a wood-fueled boiler system for heating, potentially influencing local air pollutant concentrations.

During the measurement campaign, two monitoring instruments were operated besides filter sampling. An Optical Particle Counter (OPC, Portable Aerosol Spectrometer 1.109, Grimm Aerosol Technik GmbH & Co. KG, Germany) was employed to measure PM characteristics, including dust mass fractions and particle number size distribution. The instrument operates based on laser light scattering, caused by the aerosol particles as they pass through the laser beam. The intensity of the scattered light is detected and analyzed to determine the particle size. The spectrometer is capable of detecting particles ranging from 0.25 to 32 µm in diameter (within 31 channels), with customizable settings for size range and sensitivity. This measurement method allows for continuous data collection at high (1 min) temporal resolution for the entire size range, providing real-time measurements of particle size distribution.

Black carbon concentrations in ambient particulate matter were quantified using a Portable Aethalometer (Model AE42-7, Magee Scientific, Berkeley, CA, USA). This instrument operates by continuously collecting aerosol particles on a quartz fiber filter tape and measuring the attenuation of light transmitted through the loaded filter at seven wavelengths. The instrument was run at a flow rate of 2 L/min, equipped with a PM_2.5_ size selective inlet, and raw attenuation data were corrected using the Weingartner model (Weingartner et al., 2003) for load effect correction, using mass absorption cross-section (MAC) of 16.6 m^2^/g at 880 nm, and compensation parameter (C) of 2.83. The degree of optical attenuation, resulting from the accumulation of light-absorbing particles, enables real-time determination of BC concentrations, with a high (2 min) time resolution.

Additionally, 25 PM_2.5_ aerosol samples were collected on quartz filters using a PM_2.5_ size-selective inlet, with each sample representing a 24-h collection period and an average air volume of 27 m^3^ (average airflow rate of 18.75 L/min). This procedure enabled the quantitative determination of the carbon content of the samples, performed with a thermo-optical analyzer (DRI 2015 Series 2, Aerosol Magee Scientific, Slovenia), which is particularly well-suited for determining the organic carbon (OC) and elemental carbon (EC) content of aerosol samples, facilitating source apportionment analysis.

To complement the measurements, video footage of the site and its surroundings was recorded to accurately document the operation of boilers associated with the chimneys near the measurement point. Identifying a measurement site with distinct and identifiable pollution sources presents a significant challenge in field studies. In the selected residential area, emissions from a neighboring wood-burning boiler were clearly discernible.

The nearest official air quality monitoring station operated by the Hungarian Air Quality Monitoring Network (HungaroMet, 2025a, 2025b) to the Solymár measurement site is located in the outskirts of Budapest, in the Pesthidegkút area (Fig. 1). This site is classified as an urban background station and is situated approximately 3 km to the southeast of the Solymár site. The station provides hourly resolved data on PM_10_ and PM_2.5_ mass concentrations, offering a useful reference for regional background levels in the vicinity of the study area.

### Data evaluation and processing of measurement data and samples

#### Quartz sample analysis

Quantification of the organic carbon and elemental carbon content of the 25 PM_2.5_ aerosol samples was performed following standardized thermal-optical protocol EUSAAR2 (EN 16909:2017, Cavalli et al., 2010) using DRI 2015 Series 2 OC/EC instrument (Aerosol Magee Scientific, 2022). The carbonaceous aerosol deposited on the quartz filter is thermally desorbed according to a prescribed temperature protocol, first in an inert atmosphere (helium) and then in an oxidizing atmosphere (2% oxygen, 98% helium). OC is evolved from the filter punch in a He-only (> 99.999%) atmosphere at the following temperature steps: OC1 from ambient (~ 25 °C) to 140 °C, OC2 from 140 to 280 °C, OC3 from 280 to 480 °C, and OC4 from 480 to 580 °C. EC is evolved from the filter punch in a 98% He/2% O_2_ atmosphere at the following temperature steps: EC1 at 580 °C, EC2 from 580 to 740 °C, EC3 from 740 to 840 °C, and EC4 above 840 °C. However, thermally unstable organic compounds pyrolyze (char) in the inert atmosphere to form pyrolytic carbon (PC), and if not properly accounted for, PC would be incorrectly reported as EC. To account for this, illumination by a laser beam is used to monitor the optical properties of the filter during the analysis by measuring transmittance. The time when the transmittance signal values meet the prepyrolysis value is called the OC–EC split point. For sample preparation, the ambient aerosol samples were punched three times (0.5 cm^2^ area) to prepare triplicates for analysis. The filter sections were sequentially loaded after undergoing a 90-min drying phase to remove any absorbed moisture, ensuring the accuracy of the carbon content measurements. The thermal-optical operates under the control of the LabVIEW-based software 'Carbon2015', which provides automated data acquisition and integration with hardware systems, ensuring precise calibration and consistent data processing.

#### Aethalometer model

The Portable Aethalometer utilizes a 7-wavelength measurement system that is selectively sensitive to black carbon, forming also the basis for the characterization of carbonaceous aerosol sources with high time resolution. To differentiate between black carbon originating from biomass burning (BB) and fossil fuel combustion (FF), absorption coefficients (*b_abs*) derived from the raw attenuation data and corrected for the filter loading effect were used as input for the source apportionment model described by Sandradewi et al. (2008). This so-called Aethalometer model exploits the wavelength dependence of aerosol light absorption, characterized by the Ångström exponent (*α*), calculated between 470 and 950 nm, to estimate source contributions.

In this study, we developed a customized procedure for determining the relevant Ångström exponents (*α*_*FF*_ and *α*_*BB*_). This approach assumes that the temporal variation of the BC components (BB and FF) correlates with other tracers emitted by the same sources. For instance, according to the literature (Nakkasem et al., 2024; Navinya et al., 2024), the OC1 fraction is typically associated with biomass burning, and thus, it is expected to correlate with the BB component obtained from the Aethalometer model. Similarly, the FF component, which is characteristic of fossil fuel combustion, is assumed to correlate with the OC4 fraction (Nakkasem et al., 2024, Navinya et al., 2024), corresponds to resuspended road dust. Accordingly, for the filter-based samples, the 24-h averaged OC component concentration values are paired with the corresponding 24-h averages of the BB and FF components concentration values derived from the Aethalometer data, in which the variable parameters are the *α* exponents. The Ångström exponents are therefore optimized in such a way that the selected organic carbon fractions (OC1 or OC4) show the best possible correlation with the respective black carbon components (BB and FF) derived from the Aethalometer model.

**Fig. 2:**
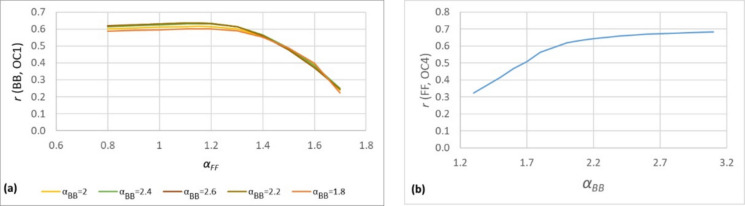
**a** Correlation coefficient between BB and OC1 as a function of the *α*_*FF*_ Ångström exponent, assuming several *α*_*BB*_. **b** Correlation coefficient between FF and OC4 as a function of the *α*_*BB*_ Ångström exponent, assuming that *α*_*FF*_ = 1.15

Thus, in the first step *α*_*FF*_ parameter was optimized to achieve the best possible correlation between BB determined by the Aethalometer model and OC1 components measured on the sampled quartz-fiber filters (Fig. 2a). As shown in Fig. 2a, for all tested *α*_*BB*_ values, the correlation coefficient (*r*) between BB and OC1 reaches its maximum at *α*_*FF*_ = 1.15.

In the following step our aim was to determine the *α*_*BB*_ parameter. For this, another well-established OC fraction was used: OC4 is known to be predominantly associated with traffic-related resuspension (Nakkasem et al., 2024). Using the previously determined *α*_*FF*_ value, we optimized the *α*_*BB*_ value that provided the strongest correlation with the OC4 measured on the filters with FF determined using the Aethalometer model. Based on the resulting curve presented in Fig. 2b, the *α*_*BB*_ value was determined to be 2.2, as the correlation exhibits a clear saturation behavior around this value, with no further meaningful improvement at higher values.

## Results and discussion

### Ambient particulate matter and fine particles

The objective of this study was to investigate periods of elevated air pollution, which are associated with potential adverse health effects and underscore the critical need for detailed source apportionment and chemical characterization. Such analyses are essential for the accurate identification of dominant local and episodic emission sources. Particular attention was given to the potential detection and attribution of emissions originating from illegal waste burning, which remains a poorly characterized yet significant contributor to ambient air pollution in certain regions.

Table 1 presents key descriptive statistics of particulate matter (PM_10_, PM_2.5_, PM_1_) and black carbon (BC) concentrations measured with high time resolution for the winter season of 2025.

**Table 1:** Statistics of PM_10_, PM_2.5_, PM_1_, and BC in 2025 with WHO guideline exceedances

Component	Min value (1 min time resolution)	Max value (1 min time resolution)	Median value (1 min time resolution)	Days exceeding WHO 24-h guideline	WHO 24-h guideline (2021)^1^	EU 24-h limit (Directive 2008/50/EC)^b^
PM_10_ (µg/m^3^)	3	208	36	16/41	45 µg/m^3^	50 µg/m^3^
PM_2.5_ (µg/m^3^)	3	205	35	39/41	15 µg/m^3^	20 µg/m^3^ (Annual) 25 µg/m^3^ (Target for 2030)
PM_1_ (µg/m^3^)	2	202	34	-	–	–
BC (µg/m^3^)	0.11	23.8	2.4	-	–	–

^a^WHO 2021 Air Quality Guidelines (PM_2.5_)

^b^EU Directive 2008/50/EC ambient air quality standards (PM_2.5_)

Out of 41 studied days, 16 days exceeded the WHO 24-h PM_10_ guideline (45 µg/m^3^), while 11 days exceeded the WHO 24-h PM_2.5_ guideline (15 µg/m^3^). PM_1_ concentrations occasionally reached very high values, up to 202 µg/m^3^; however, no WHO or EU daily guideline values exist for PM_1_. Similarly, BC levels ranged from 0.11 to 23.85 µg/m^3^, with a median of 2.407 µg/m^3^, and no official daily guideline values are established for BC.

These results clearly demonstrate frequent exceedances of health-based air quality guidelines for PM_2.5_, particularly during the colder months.

#### Background PM concentrations

To assess the background air quality, we used measurement data from the nearest suburban station (Pesthidegkút station—see Fig. 1). The impact of urban emissions is typically minor due to its location and prevailing wind directions, and it is considered an urban background station in several studies (HungaroMet, 2025a, 2025b). In our study, it thus serves excellently to examine the extent of large-scale air quality conditions, making it possible to distinguish the impact of local sources. At the Pesthidegkút station, which we refer to hereafter as the reference site, PM_2.5_ and PM_10_ are measured with hourly resolution (PM_10_ BG, PM_2.5_ BG). Unfortunately, data gaps occurred during the measurement campaigns. The similarity in the temporal patterns of concentration values measured at the two sites is examined using correlation analysis, combining the data from both measurement campaigns.

PM_10_ BG and PM_2.5_ BG concentrations at the reference site were highly correlated (r = 0.96), as well as in case of PM_2.5_ with coarse mode PM_2.5–10_ (*r* = 0.87), confirming internal consistency. However, their correlations with PM concentration at Solymár site were only moderate (PM₁₀ BG vs. PM_10_: *r* = 0.37; PM_2.5_ BG vs. PM_2.5_: *r* = 0.44), suggesting significant additional contributions from local or episodic sources such as traffic, heating, or open burning.

At the Solymár site, significantly higher concentrations were detected compared to those measured at the background station—on average 1.3 times higher for PM_10_ and twice as high for PM_2.5_. These results confirm our assumption that significant contributions from local sources can be expected at our measurement site.

#### Particulate matter concentration interrelationships

Measured PM_10_, PM_2.5_, and PM_1_ concentrations with calculated PM_1–2.5_ and PM_2.5–10_ fraction show moderate (r = 0.68 and 0.66) correlation, on average, PM_1_ accounts for 91% of the measured PM_10_ concentration, while in the case of PM_2.5_, this reaches 95%. These results indicate that the detected aerosol particles almost entirely belong to the fine fraction, which likely originates from common local combustion-related sources. At the same time, the correlation of PM₁ with both the PM_10-2.5_ coarse fraction and the PM_2.5–1_ upper accumulation-mode fraction is only around r ≈ 0.6, highlighting that the larger particles are influenced by additional, independent emission sources.

#### Visual characteristics of smoke plumes

**Fig. 3:**
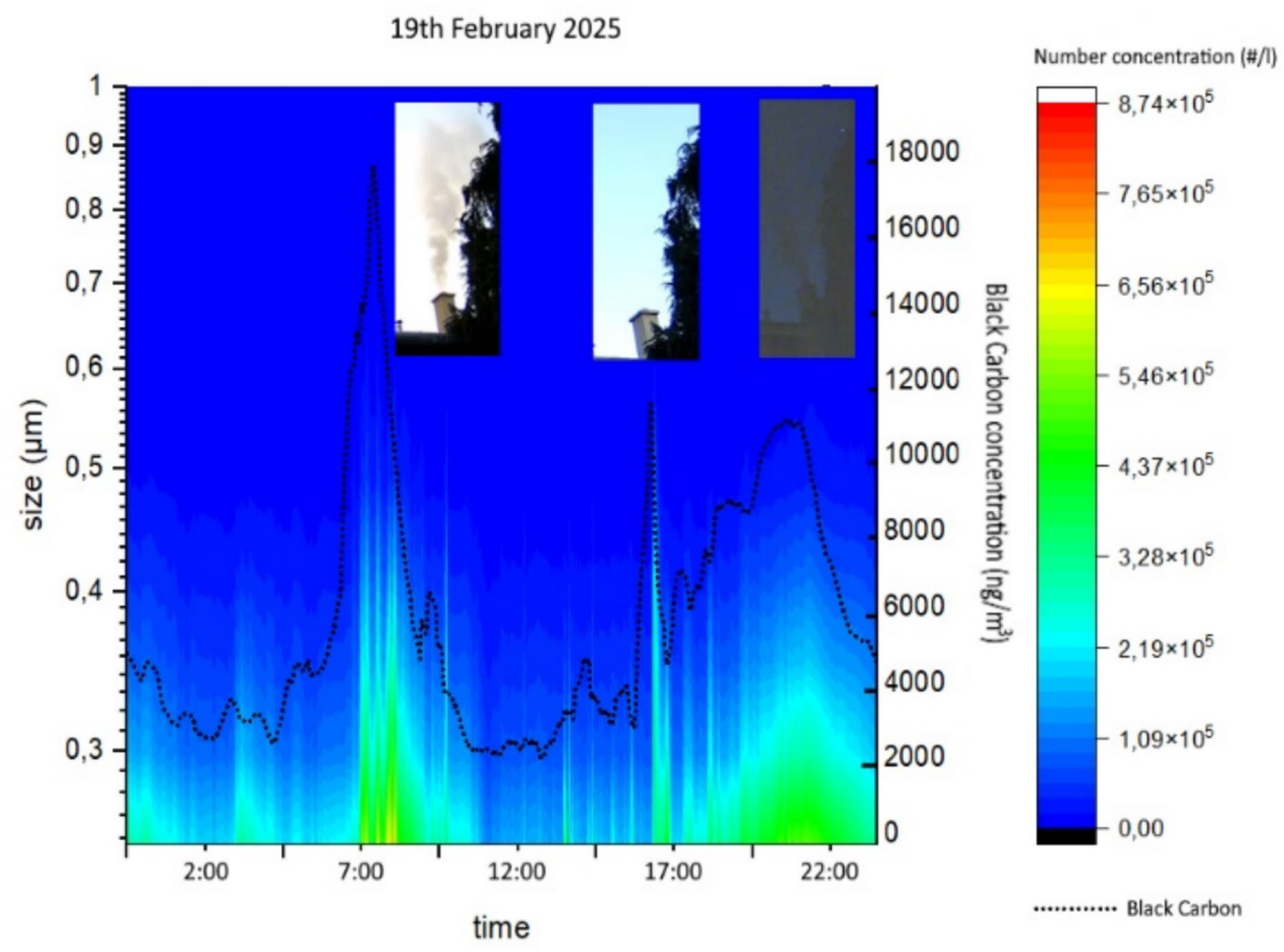
Particle size distribution and black carbon concentration on 19 February, supplemented with video snapshots selected for those periods when pronounced increases in aerosol particle concentrations were detected

To improve the characterization of local emission sources, video-based plume observations were carried out. The recordings revealed several emission episodes with visibly distinct smoke characteristics. An example of 19 February, shown in Fig. 3, emissions commenced at approximately 07:00 a.m. with light grey smoke, which progressively evolved into denser gray-brown plumes and subsequently into lighter white smoke by 09:25 a.m. Later in the afternoon (prior to 5:00 p.m.), episodic increases in both particle size distribution and BC concentrations were detected, despite the absence of visible chimney emissions. In the evening, light grey plumes were again observed, indicative of a recurrent daily activity pattern associated with local inhabitants.

Complementary particle size and black carbon measurements showed particle number concentrations peaked in the submicron range (~ 0.3–0.5 µm) despite plume color changes. BC peaks on 19 February near 07:00 a.m. and 5:00 p.m. coincided with ultrafine particle increases. The particle size distribution stayed mainly in the accumulation mode. These findings suggest plume color variations reflect combustion completeness, fuel type (including waste burning), and combustion phase, rather than particle size changes. A limitation of the present analysis is that neither the exact type nor the quantity of fuel combusted in the local stoves was known; therefore, the contribution of individual chimney emissions to the measured aerosol and BC concentrations could not be quantitatively separated. Similarly, the specific type and composition of potentially burned waste materials could not be reliably identified. Overall, although plume visibility and BC concentrations exhibited substantial temporal variability due to local emission episodes, combustion dynamics, and fuel properties, and the particle number concentrations changed significantly during the observed emission events, the particle size distribution itself remained remarkably stable throughout the measurement periods.

#### Fine particles from combustion

Based on previous studies, it is a well-known fact that particles originating from biomass burning typically fall within the 100 nm mode (Groma et al., 2022). Therefore, due to the characteristics of our measurement technique, the temporal variation in the number concentration of particles within the smallest size range (253–298 nm) measured by the OPC—i.e., the range closest to this mode—can serve as a potential tool for source identification. This study focuses on size-selected aerosols with a geometric mean diameter in the size range of 253–298 nm, representative of the accumulation mode (hereafter referred as RoAM) to capture the most pronounced biomass burning signatures within the study area. Correlation analysis reveals a strong association between RoAM particles and PM mass concentrations (*r* = 0.95 for all three components), alongside strong correlations with black carbon (BC; *r* = 0.86) and biomass burning related BC (BB; *r* = 0.86). Conversely, their weak correlation with background PM levels (*r* = 0.27–0.30) indicates limited influence from regional background aerosols and a greater sensitivity to fresh or local combustion sources. Therefore, particles in the size range of 253–298 nm serve as robust, however, not exclusive tracers for combustion-derived aerosol constituents.

### Organic and elemental carbon

In order to characterize the substantial contribution of fuel combustion to aerosol load, we conducted chemical analyses of aerosol samples. Based on this, our aim is to differentiate source contributions from various combustion processes, with focus on the detectability of illegal waste burning.

#### Correlation of TOC (total organic carbon) and TEC (total elemental carbon)

In the first stage of the analysis, we assess the total elemental carbon (TEC) and total organic carbon (TOC) content, their relative proportions, and their temporal variation. Regarding temporal trends, our objective is to investigate correlations with tracer species that may indicate common emission sources. Accordingly, we examine the correlations between individual PM components, as well as BC and BB and FF fractions, with TOC and TEC. The results of the analysis are summarized in Table 2. TOC shows a strong positive correlation with TEC (*r* = 0.89), indicating parallel changes in their absolute quantities. Since these samples represent daily (24-h) averages, the effects of highly variable emission sources (such as traffic and household combustion, which typically follow daily time patterns) cannot be studied. However, if a distinct source emerges—like occasional waste burning, it can be detected based on the differences between daily averages. As the temporal variations of the two components are strongly correlated, the subsequent discussion of the individual subcomponents (OC1–4 and EC1–4) should be presented normalized to the total component (TOC and TEC). This approach ensures that temporal changes in the total concentration do not influence the interpretation of temporal trends in the relative proportions of the subcomponents.

**Table 2:**
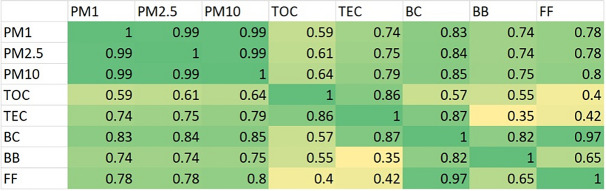
Correlation coefficients between selected aerosol components

Orthogonal regression yielded a slope between the TEC and BC measurements of 1.392 (±0.31), which is consistent with previous studies for TEC (EUSAAR2) and BC measurements. Specifically, Rigler et al. (2020) reported slopes ranging from approximately 0.44 to 1.64 depending on thermal protocols and locations, indicating variability in EC/BC relationships across environments but providing a benchmark for reliability. Our observed slope falls well within this range, supporting the reliability of our EC measurements.

As shown on. Table 2 both TOC and TEC show moderate correlation with PM_1_, PM_2.5_ and PM_10_ mass concentrations (*r* = 0.59–0.64 and 0.74–0.79 respectively), and moderate to strong correlation with BC (*r* = 0.57 and 0.87, respectively). These results indicate that the aerosol in such a complex environment the PM size fractions contain very limited information regarding emission sources.

Overall, TOC and TEC exhibit distinct but interrelated patterns, highlighting the importance of considering both fractions and their ratios for comprehensive aerosol carbon characterization.

#### TOC/TEC ratio

Previous studies have extensively discussed variations in the TOC/TEC ratio for different emission sources (Nakkasem et al., 2024), with particular emphasis on combustion-related processes; therefore, this aspect is addressed in detail in our analysis.

The TOC/TEC ratio shows correlations ranging from weakly negative to moderately positive with various factors, indicating complex interactions between organic and elemental carbon. Specifically, the TEC/TOC ratio correlates moderately with TEC (*r* = 0.80) and weakly with TOC (*r* = 0.47). This suggests that fluctuations in the TEC/TOC ratio are primarily driven by changes in TEC rather than TOC.

**Fig. 4:**
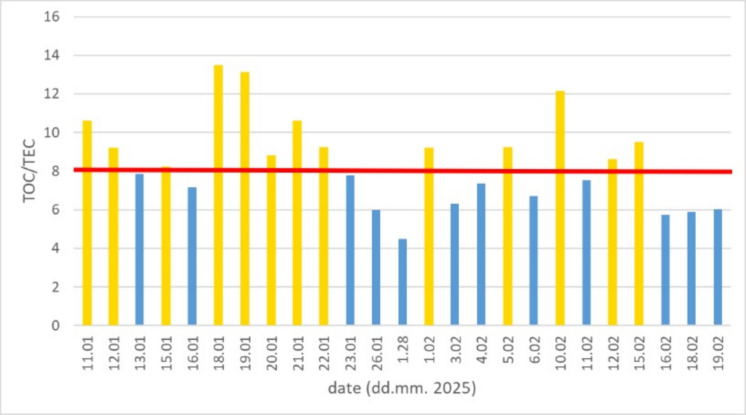
TOC/TEC ratios detected in the 24-h samples

Figure 4 shows the TOC/TEC ratios for aerosol samples. More than half of samples (marked as orange columns) exhibit TOC/TEC ratios higher than the reference value of 8 (Nakkasem et al., 2024), which is generally associated with biomass burning, whereas lower values (blue columns) typically indicate a greater influence of fossil fuel combustion. This pattern confirms that both biomass burning and traffic-related fossil fuel emission are a dominant carbonaceous source in the study area, consistent with our initial expectations. As several samples exhibit ratios well above 10, these results suggest the presence of inefficient or poor-quality combustion processes on certain days during the measurement period.

#### Elemental and organic carbon fractions

**Fig. 5:**
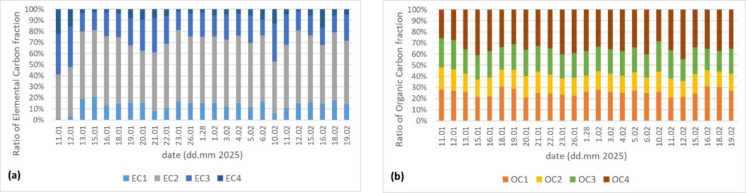
**a** Elemental carbon and** b** organic carbon fractions per sample

Figure 5 a shows the relative distribution of elemental carbon fractions (EC1, EC2, EC3, and EC4) in aerosol samples collected. Across the entire measurement period, EC2—which is generally associated with diesel exhaust emission (Nakkasem et al., 2024)—is consistently the dominant fraction, often comprising the largest proportion of total EC, while EC1 and EC3 show smaller and more variable contributions. Interestingly, there is also a notable presence of EC4 in several samples, although this component is not typically prominent in standard thermal-optical protocols.

Figure 5 b illustrates the distribution of organic carbon fractions (OC1–OC4) in aerosol samples collected. Overall, OC4 is frequently the dominant component across many samples, followed by OC2, while OC1 and OC3 contribute smaller shares.

The substantial share of OC4, which is typically associated with traffic emissions, suggests that traffic plays a significant and consistent role in the observed organic carbon load (Nakkasem et al., 2024). OC1, which is usually interpreted as a marker for biomass burning, also shows notable contributions throughout both years, while OC2, linked to coal combustion (Nakkasem et al., 2024), and OC3 appear regularly but at lower relative levels compared to OC4 and OC1.

It is important to note that these components are not conclusively linked to a single, well-defined source; therefore, they cannot be used as unambiguous markers, especially in environments with complex source mixtures. In our study, we aim to illustrate this point: while commonly accepted tracers can provide relatively accurate source characterization when only a few sources are present presumably, variations in their concentrations may serve as useful indicators of the emergence of atypical sources.

#### Sources of carbonaceous components

Based on the above, it can be concluded that the source distribution is complex, there are substantial day-to-day differences both in composition and quantity, and the identification of individual sources is challenging using only monitoring measurements.

When discussing individual organic fractions, it is advisable to analyze values normalized to the total sum in order to compare temporal trends, as this allows changes in their relative proportions to be detected. Thus, due to the significant temporal variability of the EC and OC components, their concentrations were normalized relative to TEC and TOC. Since the EC and OC fractions are normalized values, the BB and FF components used for comparison must also be normalized, which we accomplished using the corresponding BC values.

In the process of source identification, the temporal variation of each investigated parameter is examined under the assumption that components originating from the same source exhibit strong correlations.

The analysis was based on two independent datasets:

1. OC and EC components measured by DRI technique on quartz filter, and
2. BC concentrations measured by an Aethalometer. The Aethalometer model separated BC into contributions from biomass burning (BB) and fossil fuel/traffic (FF) sources.

As presented in “Aethalometer model”, the optimal values obtained for both *α*_*BB*_ and *α*_*FF*_ differ from those typically expected for pure biomass burning and fossil-fuel combustion (i.e., *α*_*FF*_ ≈ 1, whereas the fitted value is 1.15, and *α*_*BB*_ ≈ 2, whereas the fitted value is 2.2), the presence of additional, non-standard emission sources appears likely, at least intermittently. To explore this possibility, we applied a same methodology to the one presented earlier (in “Aethalometer model”), we again examine the correlation between tracers associated with the same source; however, in this step we identify those days for which the best correlation is obtained. These days are referred to as “typical days”. This approach allows the exclusion of days on which additional sources substantially affect the tracer ratios (“atypical days”).

For traffic-related emissions, the correlation between FF/BC and OC4/TOC reaches *r* = 0.64 when all sampling days are included. Restricting the analysis to the typical days—representing approximately 62% of the dataset—substantially improves the correlation to *r* = 0.87. A linear regression line was also fitted to these points.

**Fig. 6:**
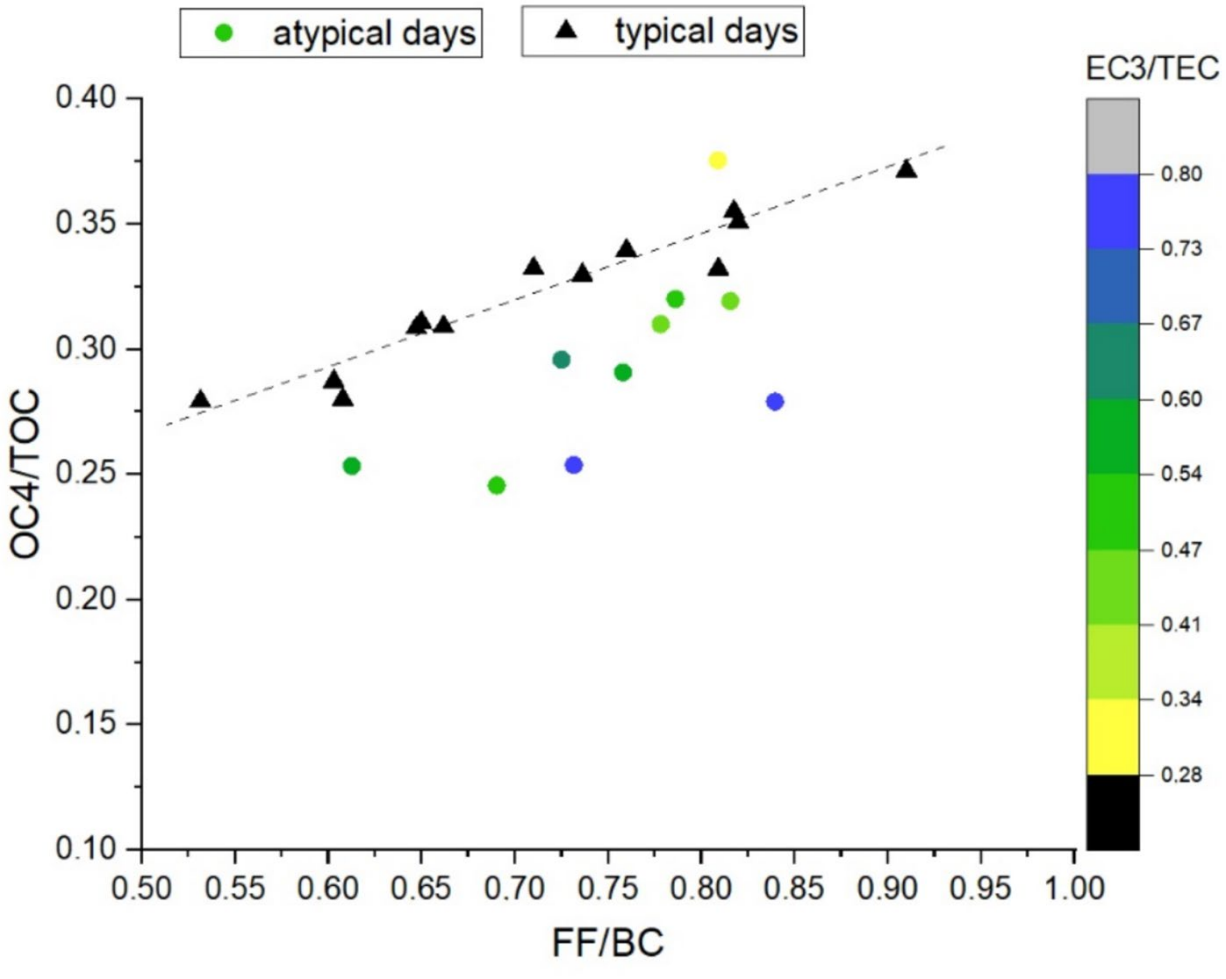
Relationship between the normalized values of traffic-related components (FF and OC4), with days characterized by purely traffic-related emissions (“typical”) shown as black triangles, and days influenced by additional sources (“atypical”) shown as colored dots. The dashed line represents the linear regression fitted to the optimal days, while the color scale indicates the EC3/TEC value determined for each data point

Next, we examined whether the atypical days are associated with other aerosol components. For each atypical day, we calculated the Euclidean distance (*dist*_*FF*_) from the regression line fitted to the typical days and investigated the relationship between *dist*_*FF*_ and additional EC and OC fractions. A strong correlation was identified between *dist*_*FF*_ and EC3/TEC (*r* = 0.89). Figure 6 shows the OC4/TOC values corresponding to FF/BC, separated into typical days (black triangles) and atypical days (colored circles). The color scale indicates the daily mean EC3/TEC values, and the dashed line represents the regression fitted to the typical days.

Because EC3 is commonly associated with coal combustion (Nakkasem et al., 2024), this result indicates that coal-related emissions are present at detectable levels, even though their contribution does not exhibit a systematic temporal pattern. This interpretation is further supported by the observation that, on the atypical days, OC4—typically linked to traffic-related resuspension—shows values lower than expected (with one exception), implying a reduced contribution of traffic emissions at similar FF levels.

Similarly, the components associated with biomass burning were examined. The relationship between BB/BC and OC1/TOC already exhibits a strong correlation when all sampling days are considered (*r* = 0.75). However, excluding approximately 43% of the days—retaining only the optimal days—further strengthens the correlation to *r* = 0.92.

**Fig. 7:**
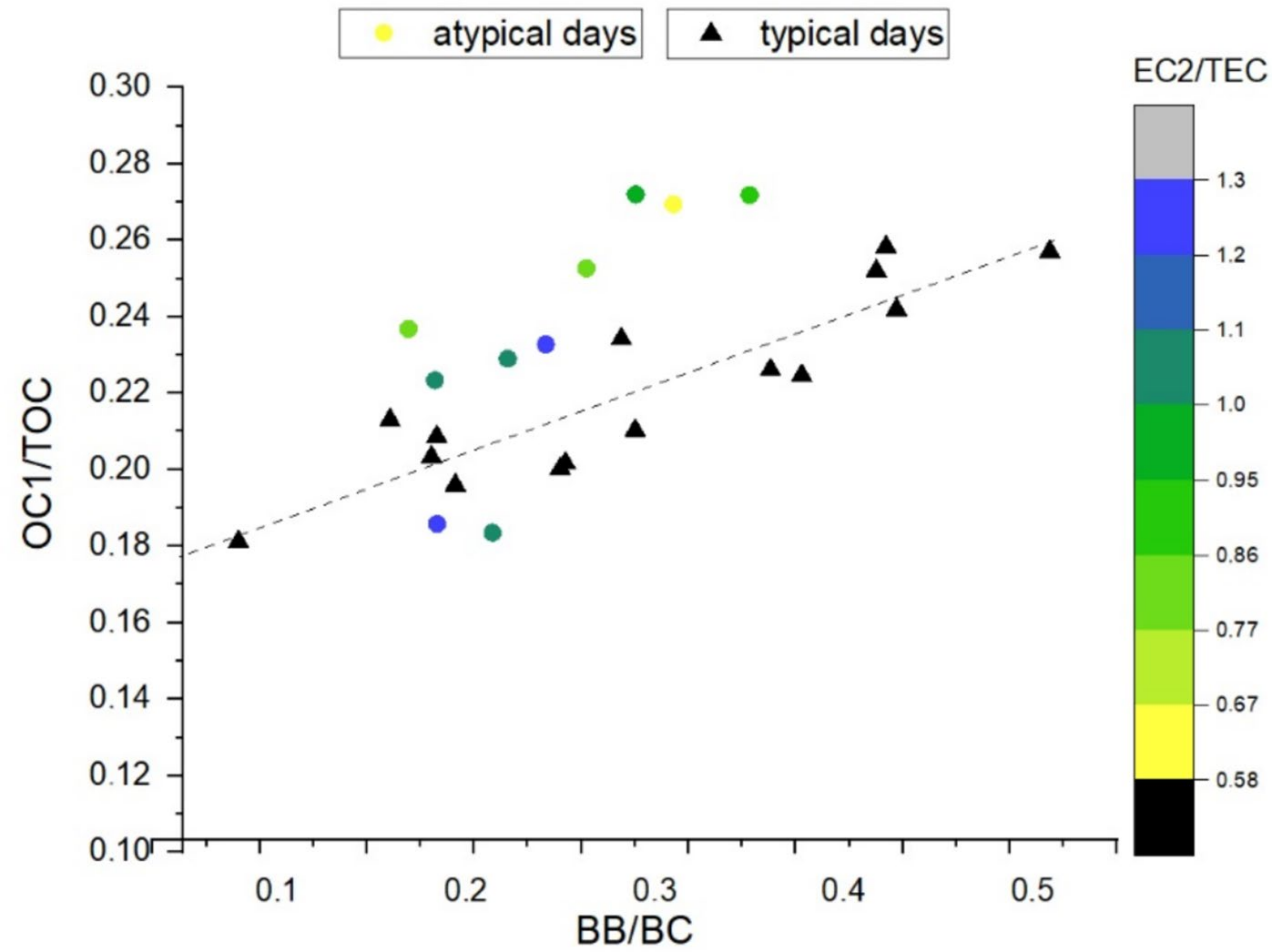
Relationship between the normalized values of biomass-burning-related components (BB and OC1), with days characterized by purely biomass-burning influence (“typical”) shown as black triangles and days affected by additional sources (“atypical”) shown as colored dots. The dashed line represents the linear regression determined for the optimal days, while the color scale indicates the EC2/TEC value associated with each measurement point

To analyze the atypical days, we applied the same procedure described above. For each atypical day, we calculated the deviation (*dist*_*BB*_) from the linear regression line fitted to BB/BC and OC1/TOC pairs using only the typical days, and evaluated the correlation of this deviation with additional EC and OC fractions. Among all OC and EC fractions, the best, however modest correlation was found between *dist*_*BB*_ and EC2/TEC (r = 0.65). Figure 7 shows the OC1/TOC values corresponding to BB/BC, separated into typical days (black triangles) and atypical days (colored circles). The color scale indicates the daily mean EC2/TEC values for each point, while the dashed line represents the regression fitted to the optimal days.

As reported by Wang et al. (2023), an increase in EC2 emission could be associated with contributions from mixed fuels and/or plastic burning. In addition, elevated OC1 levels are expected under conditions of non-pure biomass combustion or inefficient burning. Our results are consistent with this interpretation, as OC1 ratios are generally higher on atypical days compared to those with similar BB values. Although definitive evidence for waste burning cannot be established, which is not surprising, given that the amount of household waste available for combustion is typically small relative to the quantity of fuel normally burned.

## Conclusion

Using plastic, rubber and other waste as a fuel for heating is still a frequent activity in many locations of the world. Laboratory experiments introduced in the literature provide some insight into the types of pollutant emissions associated with improper household combustion; however, they are sparse, especially related to particle composition characterization. In this study, conventional monitoring tools were complemented by aerosol organic composition analysis to identify components contributing to the heavily polluted winter atmosphere of a small urban area. Although no single component could be regarded as a definitive marker, our results suggest that atypical or inefficient combustion events can be identified. Temporal trend analysis of various carbonaceous components provided multiple lines of evidence suggesting poor-quality stove operation and the illegal burning of waste. Detailed mapping of this phenomenon would help target regions where public awareness and education should be prioritized, as this is the most effective way for inhabitants to contribute to improving local air quality.

## Data Availability

Data is available upon request.

## References

[CR1] Aerosol d.o.o. / Magee Scientific. (2022). *DRI 2015 Series 2 laboratory OC/EC analyzer user’s manual (Version 0.2.6)*. Aerosol d.o.o.

[CR2] Brooks, A. W., Sandri, B. J., Nixon, J. P., Nurkiewicz, T. R., Barach, P., Trembley, J. H., Boles, J. C., Buxbaum, J. D., Norden, D. M., Nelson, L. H., & Lo, D. D. (2024). Neuroinflammation and brain health risks in veterans exposed to burn pit toxins. *International Journal of Molecular Sciences,**25*(18), Article Article 9759. 10.3390/ijms25189759 39337247 10.3390/ijms25189759 PMC11432193

[CR3] Cavalli, F., Viana, M., Yttri, K. E., Genberg, J., & Putaud, J.-P. (2010). Toward a standardised thermal-optical protocol for measuring at-mospheric organic and elemental carbon: The EUSAAR protocol. *Atmospheric Measurement Techniques,**3*, 79–89. 10.5194/amt-3-79-2010

[CR4] Cohen, A. J., Brauer, M., Burnett, R., Anderson, H. R., Frostad, J., Estep, K., Balakrishnan, K., Brunekreef, B., Dandona, L., Dandona, R., Feigin, V., Freedman, G., Hubbell, B., Jobling, A., Kan, H., Knibbs, L., Liu, Y., Martin, R., & Forouzanfar, M. H. (2017). Estimates and 25-year trends of the global burden of disease attributable to ambient air pollution: An analysis of data from the Global Burden of Disease Study 2015. *The Lancet,**389*(10082), 1907–1918. 10.1016/S0140-6736(17)30505-6 PMC5439030 28408086

[CR5] European Commission. (2021). Zero Pollution Action Plan: Towards zero pollution for air, water and soil. https://environment.ec.europa.eu/strategy/zero-pollution-action-plan_en.

[CR6] European Commission. (2023). Circular economy action plan. https://environment.ec.europa.eu/strategy/circular-economy-action-plan_en.

[CR7] European Environment Agency (EEA), (2023a). Many EU Member States not on track to meet recycling targets for municipal waste and packaging waste (Briefing No. 28/2022). https://www.eea.europa.eu/publications/many-eu-member-states.

[CR8] Eurostat. (2018, January 23). 480 kg of municipal waste generated per person in the EU. https://ec.europa.eu/eurostat/web/products-eurostat-news/-/DDN-20180123-1.

[CR9] Forouzanfar, M. H., Afshin, A., Alexander, L. T., Anderson, H. R., Bhutta, Z. A., Biryukov, S., et al. (2015). Global, regional, and national comparative risk assessment of 79 risks for 188 countries, 1990–2013: A systematic analysis for the Global Burden of Disease Study 2013. *The Lancet,**386*(10010), 2287–2323. 10.1016/S0140-6736(15)00128-2 PMC4685753 26364544

[CR10] Gallup, 2024 https://news.gallup.com/poll/649931/adults-worldwide-live-households-burn-waste.aspx

[CR11] Gonçalves, C., Alves, C., Fernandes, A. P., Monteiro, C., Tarelho, L., Evtyugina, M., & Pio, C. (2012). Organic compounds in PM2.5 emitted from fireplace and woodstove combustion of typical Portuguese wood species. *Atmospheric Environment,**45*(26), 4533–4545. 10.1016/j.atmosenv.2011.05.071

[CR12] Groma, V., Alföldy, B., Börcsök, E., Czömpöly, O., Füri, P., Horváthné Kéri, A., Kovács, G., Török, S., & Osán, J. (2022). Sources and health effects of fine and ultrafine aerosol particles in an urban environment. *Atmospheric Pollution Research,**13*(2), Article Article 101302. 10.1016/j.apr.2021.101302

[CR13] Health Effects Institute. (2013). Understanding the health effects of ultrafine particles. https://www.healtheffects.org/publication/understanding-health-effects-ultrafine-particles.

[CR14] Hoffer, A., Kiss, G., & Tóth, Á. (2020). Emission factors for indoor combustion of municipal waste. *Environmental Pollution,**264*, 113–130.

[CR15] HungaroMet (2025). Expected evolution of air pollution . https://legszennyezettseg.met.hu/en/.

[CR16] HungaroMet (2025). Automatic air quality monitoring network. https://legszennyezettseg.met.hu/en/air-quality/automata-meroallomasok.

[CR17] Kantar Hoffmann Company. (2017). Household waste burning practices in Hungary.

[CR18] Kantar Hoffmann Company. (2020). Assessment of household waste burning in Hungary.

[CR19] Karagulian, F., Van Dingenen, R., Belis, C. A., Janssens-Maenhout, G., Crippa, M., Guizzardi, D., & Dentener, F. (2015). Contributions to cities’ ambient particulate matter (PM): A systematic review of local source contributions at global level. *Atmospheric Environment,**120*, 475–483. 10.1016/j.atmosenv.2015.08.087

[CR20] Kilian, J., & Kitazawa, M. (2018). The emerging risk of exposure to air pollution on cognitive decline and Alzheimer’s disease. *Biomedical Journal,**41*(3), 141–162. 10.1016/j.bj.2018.06.001 30080655 10.1016/j.bj.2018.06.001 PMC6138768

[CR21] Kreyling, W. (2003). Toxicokinetics of inhaled nanoparticles. In *Nanomaterials* (pp. 32–36). Viley-VCH. 10.1002/9783527673919.ch3

[CR22] Manisalidis, I., Stavropoulou, E., Stavropoulos, A., & Bezirtzoglou, E. (2020). Environmental and health impacts of air pollution: A review. *Frontiers in Public Health,**8*, Article 14. 10.3389/fpubh.2020.00014 32154200 10.3389/fpubh.2020.00014 PMC7044178

[CR23] Nakkasem, K., Thepanondh, S., Yabueng, N., & Chantara, S. (2024). Organic and elemental carbon characteristics in PM2.5 across diverse landscapes. Mahidol University & Chiang Mai University.

[CR24] Navinya, C., Kapoor, T. S., Anurag, G., Venkataraman, C., Phuleria, H. C., & Chakrabarty, R. K. (2024). *Brownness* of organics in anthropogenic biomass burning aerosols over South Asia. *Atmospheric Chemistry and Physics,**24*, 13285–13297. 10.5194/acp-24-13285-2024

[CR25] Pastorello, C., Caserini, S., Galante, S., Dilara, P., & Galletti, F. (2011). Importance of activity data for improving the residential wood combustion emission inventory at regional level. *Atmospheric Environment,**45*(17), 2869–2876. 10.1016/j.atmosenv.2011.02.070

[CR26] Rigler, M., Drinovec, L., Lavrič, G., Vlachou, A., Prévôt, A. S. H., Jaffrezo, J. L., Stavroulas, I., Sciare, J., Burger, J., Kranjc, I., Turšič, J., Hansen, A. D. A., & Močnik, G. (2020). The new instrument using a TC-BC (total carbon-black carbon) method for the online measurement of carbonaceous aerosols. *Atmospheric Measurement Techniques,**13*(8), 4333–4351. 10.5194/amt-13-4333-2020

[CR27] Sandradewi, J., Prevot, A. S. H., Szidat, S., Perron, N., Alfarra, M. R., Lanz, V. A., Weingartner, E., & Baltensperger, U. (2008). Using aerosol light absorption measurements for the quantitative determination of wood burning and traffic emission contributions to particulate matter. *Environmental Science & Technology,**42*(9), 3316–3323. 10.1021/es702253m 18522112 10.1021/es702253m

[CR28] Századvég Foundation. (2018). Study on household waste burning and its implications for air quality in Hungary.

[CR29] Tiwari, S., Bisht, D. S., & Srivastava, M. K. (2015). Impact of meteorology and atmospheric pollutants on visibility: A case study from Delhi, India. *Atmospheric Pollution Research,**6*(4), 552–560.

[CR30] Wang, X., Firouzkouhi, H., Chow, J. C., Watson, J. G., Ho, S. S. H., Carter, W., & De Vos, A. S. M. (2023). Chemically speciated air pollutant emissions from open burning of household solid waste from South Africa. *Atmospheric Chemistry and Physics,**23*, 15375–15393. 10.5194/acp-23-15375-2023

[CR31] Weingartner, E., Saathoff, H., Schnaiter, M., Streit, N., Bitnar, B., & Baltensperger, U. (2003). Absorption of light by soot particles: Determination of the absorption coefficient by means of aethalometers. *Journal of Aerosol Science,**34*(10), 1445–1463. 10.1016/S0021-8502(03)00359-8

[CR32] World Health Organization. (2021). WHO global air quality guidelines: PM2.5, PM10, ozone, nitrogen dioxide, sulfur dioxide and carbon monoxide. https://apps.who.int/iris/handle/10665/345329. 34662007

[CR33] World Health Organization. (2024a). Chronic obstructive pulmonary disease (COPD). https://www.who.int/news-room/fact-sheets/detail/chronic-obstructive-pulmonary-disease-(copd).

[CR34] World Health Organization. (2024b). Asthma. https://www.who.int/news-room/fact-sheets/detail/asthma.

[CR35] World Health Organization. (2024c). *Household air pollution and health*.

